# Bone marrow edema of the medioplantar talar head is associated with severe ligamentous injury in ankle sprain

**DOI:** 10.1007/s00256-022-04043-3

**Published:** 2022-03-31

**Authors:** Tina Passon, Christoph Germann, Benjamin Fritz, Christian Pfirrmann, Reto Sutter

**Affiliations:** Department of Radiology, Balgrist University Hospital, Forchstrasse 340, CH-8008 Zurich, Switzerland

**Keywords:** Magnetic resonance imaging, Bone marrow, Edema, Ankle injuries, Talus

## Abstract

**Purpose:**

To investigate the predictive value of talar head edema (THE) in acute ankle sprain for the presence of concomitant ligament injuries.

**Methods:**

This retrospective study was approved by the ethics committee and informed consent was obtained. One hundred patients (mean age: 37 years ± 14 [standard deviation], range 13–77 years) with MRI of the ankle after acute trauma were included. The cohort in this matched-pair study consisted of 50 patients with THE (group 1) and 50 patients without THE (group 2). Two readers independently evaluated presence and size of bone marrow edema of the talus head and injuries of the lateral, medial, talonavicular, and spring ligament complex. Statistics included intraclass correlation coefficient (ICC) and Kappa statistics as well as parametric and non-parametric tests.

**Results:**

On average, patients with THE demonstrated significantly more ligament injuries in comparison to patients without THE (3.7 vs. 1.3, *p* ≤ 0.01). Also, in patients with THE, the number of injured ligaments was significantly higher at the lateral (*p* = 0.03), medial (*p* ≤ 0.01), and talonavicular (*p* ≤ 0.01) compartment in comparison to patients without THE. The most frequently injured ligaments in patients with THE were the anterior talofibular ligament (60%) and the anterior tibiotalar ligament (42%). There was no significant correlation between edema size and the number of injured ligaments or compartments (*p* = 0.5).

**Conclusion:**

THE is associated with more extensive ligamentous ankle injury, in particular to the medial and lateral collateral ligament complex, and therefore indicative of severe ankle trauma.

**Supplementary Information:**

The online version contains supplementary material available at 10.1007/s00256-022-04043-3.

## Introduction

Acute injuries of the ankle are among the most common injuries of the musculoskeletal system. In particular, affection of soft tissues is often seen [1, 2]. The most common mechanism of ankle ligament injury is inversion force of the ankle with the foot in plantarflexion [3].

Lateral collateral ligament injuries occur with an incidence of 0.9/1000 athlete exposures [4]. Data from emergency departments in the USA suggest an incidence rate of 2–7 acute ankle sprains/1000 person-years in the general population [4].

The classic cascade of tearing is anterior talofibular, calcaneofibular, and last posterior talofibular ligament [5]. But only few patients have isolated lateral ligament complex injuries. In most cases, there are other associated findings like bone marrow edema, which was seen in 76% of patients with lateral ligament injuries [6]. While bone marrow edema is common in the posttraumatic ankle and is usually related to bone contusion or avulsion fractures, data about the specific location or patterns of bone marrow edema is lacking [6; 7].

The majority of patients who undergo MRI after an ankle sprain show a combination of ligamentous injuries and bone marrow edema [6].

Bone marrow edema secondary to bone impaction or bone contusion is thought to represent trabecular microcontusions and fractures, edema, hemorrhage, or reaction after stress [6; 7].

When reporting MRI examinations of patients with ankle trauma in our hospital, we often noticed talar bone marrow edemas of similar location and pattern, located at the medioplantar aspect of the talar head. Only few studies evaluated these findings and reports on associations with ligamentous injuries of the ankle are missing so far.

Especially, there is few data whether this isolated bone marrow edema correlates with the severity of injury. Therefore, the purpose of this study was to investigate the occurrence of THE in patients with ankle injuries and evaluate a possible association with ankle ligament injuries.

## Materials and methods

This retrospective study was approved by the local ethics committee.

### Subjects

**Fig. 1:**
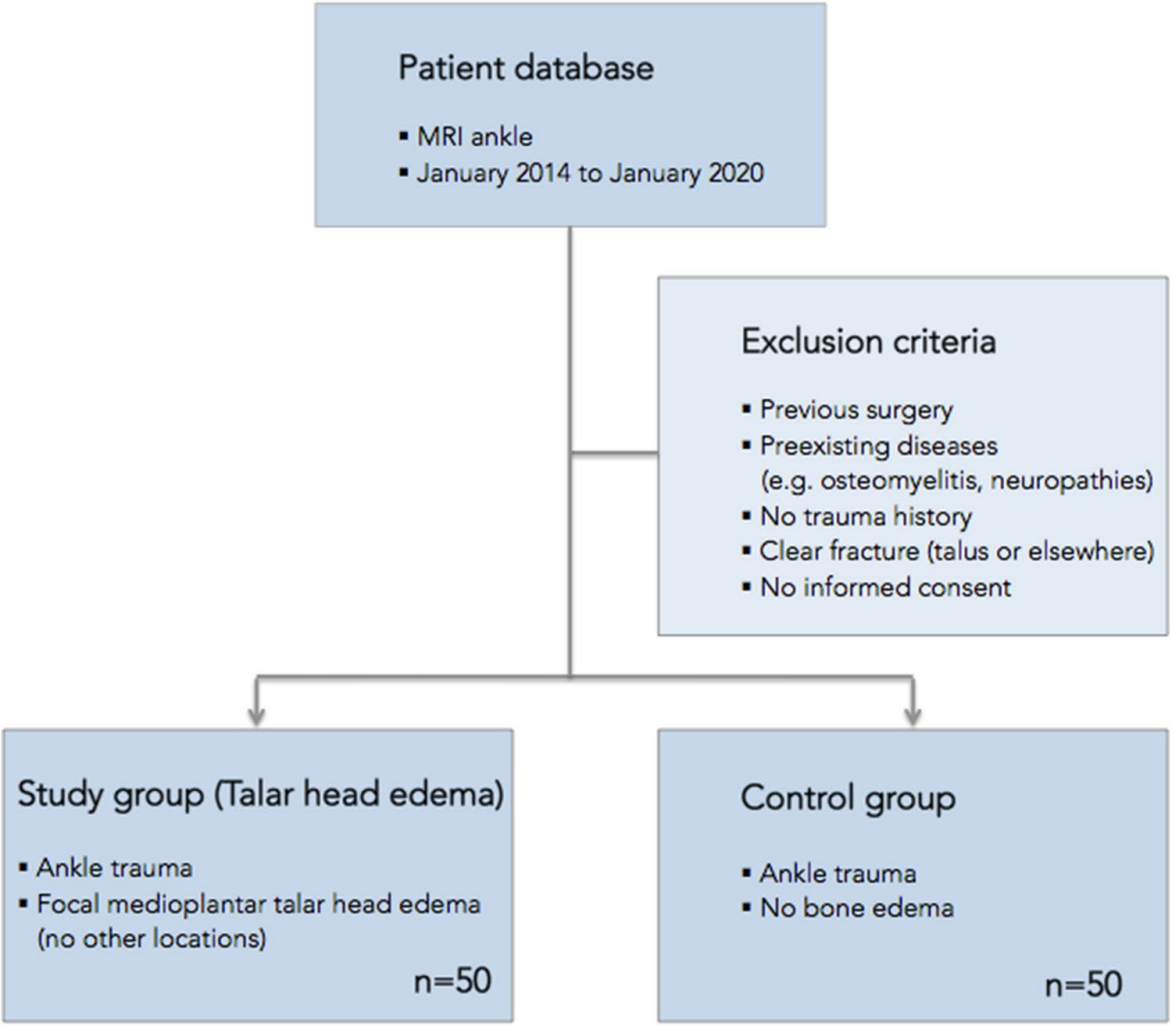
Flowchart with criteria for patient inclusion and exclusion

We performed a retrospective database search using our radiological information system (RIS) and picture archiving and communication system (PACS) to search patient data collected from January 2014 to January 2020. Inclusion criteria were focal bone marrow edema of the talus head and dedicated ankle MRI examination performed in our institution after ankle trauma. There was no bone marrow edema in other locations of the foot. Exclusion criteria for this study were previous foot surgery (e.g., ligamentoplasty, debridement, metal implants), patients with preexisting diseases (ankle tumors, systemic inflammatory or infectious disease, neuropathic joints), no history of trauma, and clear fractures (talus or elsewhere). Fifty consecutive patients with THE were retrospectively selected (36 women, 14 men; mean age: 37.7 years ± 13.4 standard deviation (SD)) suffering from pain after twisted ankle (Fig. 1).

As controls, 50 age- and gender-matched clinical patients who also suffered from ankle trauma and underwent dedicated ankle MRI without displaying bone marrow edema of the medioplantar head were consecutively included. The control group consisted of 30 women and 20 men (mean age: 36.7 years ± 14.8 SD). Due to matching, gender proportions and age were not significantly different between the study and control group (*p* = 0.29 and *p* = 0.73, respectively). In about one third of the cases (14 patients study group and 19 patients control group), there was no exact documentation of the date of trauma, 16 patients (9 patients study group and 7 patients control group) had an ankle sprain less than 1 week ago, 21 patients (8 patients study group and 13 patients control group) had trauma 1 to 6 months ago, and 28 patients (15 patients study group and 13 patients control group) 6 to 12 months ago.

### MR imaging protocol

MR imaging was acquired as part of the clinical routine workup in patients with ankle sprains.

MRI examinations were performed on a clinical 1.5-T or 3-T unit (Avanto-fit or Skyra-fit, Siemens Healthcare Erlangen, Germany) and a 16-channel bootshaped foot/ankle coil using our standard trauma protocol for ankle/hindfoot imaging. This MRI protocol consists of the following sequences: a sagittal short tau inversion recovery (STIR) sequence, a coronal and axial proton density-weighted sequence acquired in Dixon-technique with in-phase and water-only images, and a transverse oblique T1-weighted sequence with perpendicular angulation to the long axis of the peroneal and flexor tendons. The image parameters were as follows, with slight variations between the two field strengths: STIR sag (repetition time TR, 4000–4270 ms; echo time TE, 31–37 ms; field-of-view FOV, 170 mm; matrix, 384; slice thickness, 3 mm), Cor PD fs (TR 3700–4630 ms; TE 39–43 ms; FOV 160 mm; matrix 448–512; slice thickness 3 mm), Tra PD fs (TR 3700–4630 ms; TE 39–43 ms; FOV 160 mm; matrix 448–512; slice thickness 4 mm), T1 tra oblique (TR 400–500 ms; TE 10–13 ms; FOV 160 mm; matrix 512; slice thickness 4 mm).

### MR imaging analysis

Two board-certified and fellowship-trained specialized musculoskeletal radiologists (each with two years of experience), blinded to all clinical information, independently analyzed MR images of group 1 and 2 on a dedicated PACS workstation in a randomized fashion.

**Fig. 2:**
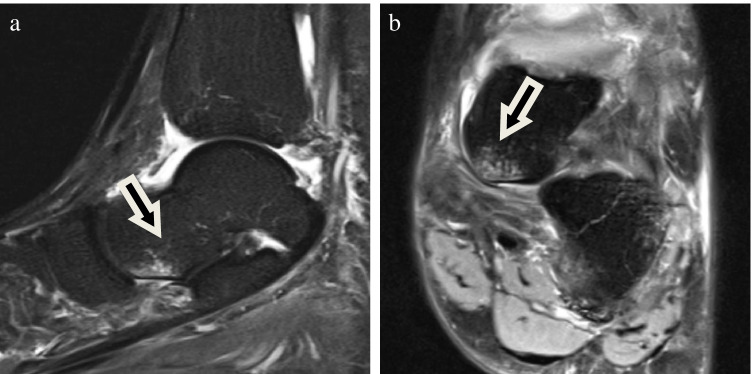
Forty-three-year-old man with ankle sprain 6 days before imaging. Sagittal short tau inversion recovery sequence (**a**) and coronal (**b**) proton density fat saturated images showed small bone marrow edema of the medioplantar head of the talus (arrows) and associated rupture of superomedial ligament of spring ligament complex

**Fig. 3:**
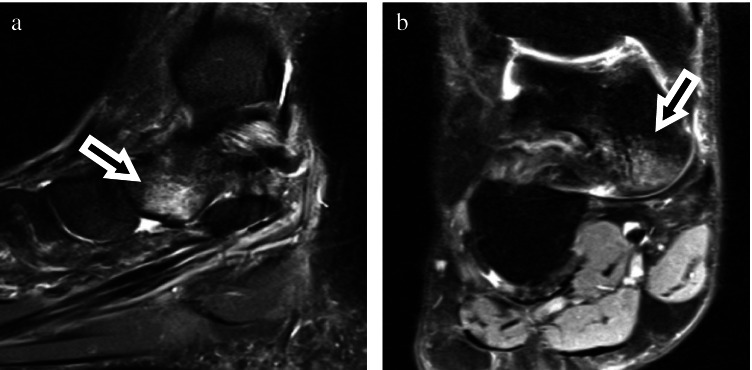
Thirty-seven-year-old woman with pain after ankle sprain. Sagittal short tau inversion recovery sequence (**a**) and coronal (**b**) proton density fat saturated images showed moderate bone marrow edema of the medioplantar head of the talus (arrows)

All cases with bone marrow edema in the medioplantar aspect of the talar head were selected. Bone marrow edema was defined as a diffuse trabecular hyperintensity on STIR and fat-suppressed PD-weighted sequences without clear demarcation of a fracture line. Only bone marrow edema pattern which was clearly located at the medioplantar portion of the talus head and with extension to the cortical bone qualified to represent THE. The size of the THE was measured in all three planes and the ellipsoid formula (*V* = 4/3 × *π* × *a* × *b* × *c*) was used to obtain the bone marrow edema volume in mm^3^ (Figs. 2 and 3).

Ankle ligament sprains were graded on the basis of the extent of fiber disruption and signal intensity on STIR and PD-weighted sequences as given in Table 1 [7]. The classification included accordingly three grades: normal (grade 0), sprain or partial rupture (grade 1), and complete rupture (grade 2) with mostly ruptured fibers.

**Table 1:** Ankle ligament sprains were graded on the basis of the extent of fiber disruption and signal intensity on short tau inversion recovery sequence and proton density fat saturated sequences

Grade	Description	Ligament appearance
*0*	*Normal*	Hypointense signal with normal thickness and without fiber discontinuity
*1*	*Sprain or partial rupture*	Hyperintense signal and/or fiber disruption of 0 to 80%
*2*	*Complete rupture*	Fiber disruption of 80 to 100%

For the analysis, the ligaments were divided into four compartments: lateral (anterior talofibular, calcaneofibular, posterior talofibular ligament), medial (tibionavicular, tibiospring, tibiocalcaneal, anterior tibiotalar, posterior tibiotalar ligament), spring ligament complex (superomedial calcaneonavicular, medioplantar oblique, inferoplantar longitudinal ligament), and talonavicular compartment (talonavicular ligament). Each ligament was analyzed separately.

### Statistical analysis

For all statistic calculations, a statistical software (IBM SPSS Statistics for Windows, Version 23.0, Armonk, NY) was used. Inter-reader agreement was calculated using intraclass correlation coefficient (ICC) and *κ* statistics. Agreement was interpreted as slight (*κ*, 0–0.20), fair (*κ*, 0.21–0.40), moderate (*κ*, 0.41–0.60), substantial (*κ*, 0.61–0.80), or excellent (*κ*, 0.81–1.00) [8]. For categorical evaluations of the ligamentous findings and gender distribution, the Chi-squared-test was applied to analyze differences between groups. Fisher’s exact test was used to determine if there are nonrandom associations between the presence of edema and ligamentous injury. Mann Whitney *U*-test was used to determine the significant difference between the number of ligamentous injuries with edema and without edema. Spearman’s rank-order correlation was used to assess the strength and direction of associations between number of ligamentous injuries and size of edema. A *p* value of < 0.05 was defined to indicate significant differences.

## Results

### Ligament injuries

#### Lateral collateral ligament injuries

**Fig. 4:**
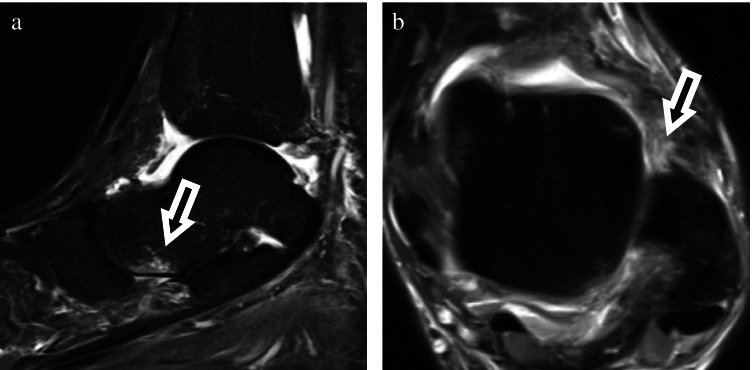
Forty-three-year-old man with ankle sprain 6 days before imaging. Capital short tau inversion recovery sequence (**a**) and axial (**b**) proton density fat saturated images showed small bone marrow edema of the medioplantar talar head (**a**) and rupture of the anterior talofibular ligament (**b**)

In the group with THE, there were significantly more tears of the anterior talofibular ligament (ATFL) than in the control group (*p* = 0.01), with 8 patients with ATFL sprain and 22 patients with ATFL tear **(**Fig. 4**)** vs. 1 patient with ATFL sprain and 17 patients with ATFL tear in the control group.

#### Medial collateral ligament injuries

In the group with THE, there were significantly more tears of the tibionavicular ligament (*p* ≤ 0.01), with 13 patients with sprain and six patients with complete tear vs. 2 patients with sprain and 1 patient with tear in the control group.

Also the values for the tibiospring ligament (TSL) showed statistically significant differences, with more tears in the THE group (*p* = 0.007). There were 16 patients with sprain of the TSL and 2 patients with rupture within the THE group and 5 patients with sprain of the TSL and no patients with rupture within the patients without THE.

**Fig. 5:**
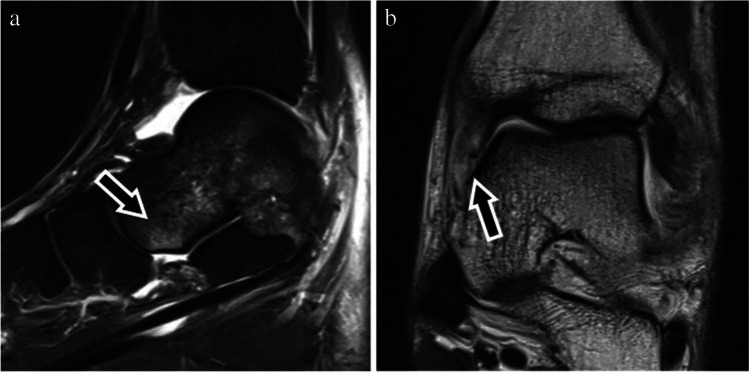
Thirty-four-year-old man with ankle sprain one week before imaging. Sagittal short tau inversion recovery sequence (**a**) and coronal (**b**) proton density images showed small bone marrow edema of the medioplantar talar head (**a**) and rupture of the anterior tibiotalar ligament (**b**)

In the group with THE, there were significantly more tears of the anterior tibiotalar ligament (aTTL) (*p* ≤ 0.01), with 17 patients with sprain and four patients with tear **(**Fig. 5**)** vs. 6 patients with sprain and no patients with tear in the control group.

Furthermore, there were statistically significant differences between both groups regarding injury of the posterior tibiotalar ligament (pTTL) (*p* = 0.04). Fourteen patients showed sprain and only 1 patient had a rupture of the pTTL in the THE group. There were 5 patients with sprain and no patient with tear in the control group.

#### Spring ligament complex injuries

The superomedial calcaneonavicular ligament (smCNL) of the spring ligament complex showed significant differences, with 12 patients with sprain (*p* = 0.01) and THE and just 2 patients with a sprain of the smCNL without THE. There was no complete tear in the THE group or the control group.

Regarding the inferoplantar longitudinal calcaneonavicular ligament (iplCNL), there was also a significant difference (*p* = 0.03). There were 10 patients with iplCNL sprain in the THE group and 40 patients with an intact ligament. In the control group, there were 2 patients with iplCNL sprain and 48 patients with a normal ligament. A complete tear was diagnosed in neither of the groups.

#### Talonavicular injuries

**Fig. 6:**
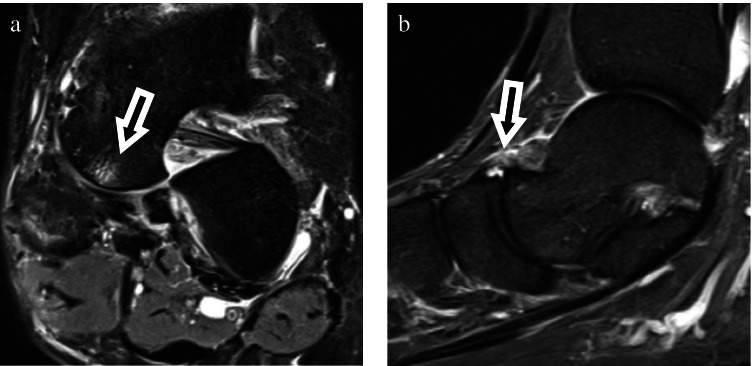
Fifty-four-year-old man with pain after ankle sprain. Coronal proton density fat saturated (**a**) and sagittal short tau inversion recovery sequence (**b**) images show small bone marrow edema of the medioplantar head (**a**) and rupture of the talonavicular ligament (**b**)

There were 13 sprains and 1 tear of the talonavicular ligament (TNL) in the THE group, while 36 patients showed no sprain or tear (Fig. 6) of the talonavicular ligament.

In the control group, the talonavicular ligament was always normal and the differences between the THE group and the control group were statistically significant (*p* ≤ 0.01).

##### Correlation between number of ligament injuries and the presence of edema

Compared to the control group, patients with THE had significantly more ligamentous injuries (sprain and rupture) of the lateral and medial compartment, as well as the talonavicular ligament (Table 2). Injuries of the talonavicular ligament were exclusively seen in patients with THE. Interestingly, injuries of the spring ligament complex were less common in patients with THE, but without statistical significance.

**Table 2:** Numbers of patients with ligament injuries after ankle sprains in the group with talar head edema and the control group. Percentages in parentheses

Number of patients with ligament injuries			*p* value
	Study group	Control group	
*Lateral compartment*	30 (60%)	18 (36%)	*p* = 0.03
*Medial compartment*	28 (56%)	10 (20%)	*p* ≤ 0.01
*Spring ligament complex*	14 (28%)	26 (52%)	*p* = 0.08
*Talonavicular ligament*	14 (28%)	0 (0%)	*p* ≤ 0.01

#### Combination of ligament injuries

In the group of patients with THE, there were statistically significantly more combination injuries in the majority of comparisons (*p* ≤ 0.01–0.03), except for the following two combinations: lateral complex with spring ligament complex (*p* = 0.41), and lateral complex with medial complex and spring ligament complex (*p* = 0.07) (Table 3). The combination of injured medial complex and lateral complex was the most common association, and was found in 38% in the group with THE, versus 16% in the control group (*p* = 0.02).

**Table 3:** Combination of ligament injuries: number of patients (study group and control group) who injured a combination of two or more ligaments. Percentages in parentheses

Combination of ligament injuries	Study group	Control group	*p*value
*LC* + *MC*	19 (38%)	8 (16%)	*p* = 0.02
*LC* + *SLC*	10 (20%)	6 (12%)	*p* = 0.41
*LC* + *TNL*	11 (22%)	0 (0%)	*p* ≤ 0.01
*MC* + *SLC*	12 (24%)	3 (6%)	*p* = 0.03
*MC* + *TNL*	10 (20%)	0 (0%)	*p* ≤ 0.01
*SLC* + *TNL*	9 (18%)	0 (0%)	*p* ≤ 0.01
*LC* + *MC* + *SLC*	10 (20%)	3 (6%)	*p* = 0.07
*LC* + *MC* + *TNL*	9 (18%)	0 (0%)	*p* ≤ 0.01
*LC* + *SLC* + *TNL*	9 (18%)	0 (0%)	*p* ≤ 0.01
*MC* + *SLC* + *TNL*	9 (18%)	0 (0%)	*p* ≤ 0.01
*LC* + *MC* + *SLC* + *TNL*	7 (14%)	0 (0%)	*p* = 0.02

#### Numbers of injured ligaments within the two groups

There were significantly more ligaments injured within the THE group than in the group without bone marrow edema (*p* ≤ 0.01): 3.7 (mean, ± 3.7 SD) ligaments were injured in the THE group, vs. 1.3 (mean, ± 2.0 SD) ligaments in the group without THE.

Regarding the number of injured compartments, patients with THE showed a significantly higher number of injured compartments with 1.7 (mean, ± 1.3 SD) than patients without THE with 0.7 (mean, ± 1.1 SD) (*p* ≤ 0.01).

An overview of all ligament injuries and numbers of patients is given in Table 4.

**Table 4:** Overview of all ligament injuries, numbers of patients and percentages (in parentheses) in the group with talar head edema and the control group, and *p* values

	Study group: Talar head edema			Control group: No talar head edema			*p* value
	*0 (normal)*	*1 (sprain)*	*2 (tear)*	*0 (normal)*	*1 (sprain)*	*2 (tear)*	
*Lateral compartment*							
*ATFL*	*20/50 (40%)*	*8/50 (16%)*	*22/50 (44%)*	*32/50 (64%)*	*1/50 (2%)*	*17/50 (34%)*	*0.01*
*CFL*	*28/50 (56%)*	*8/50 (16%)*	*14/50 (28%)*	*35/50 (70%)*	*7/50 (14%)*	*8/50 (16%)*	*0.3*
*PTFL*	*45/50 (90%)*	*5/50 (10%)*	*0*	*47/50 (94%)*	*3/50 (6%)*	*0*	*0.7*
*Medial compartment*							
*TN*	*31/50 (62%)*	*13/50 (26%)*	*6/50 (12%)*	*47/50 (94%)*	*2/50 (4%)*	*1/50 (2%)*	≤ 0.01
*TSL*	*32/50 (64%)*	*16/50 (32)%*	*2/50 (4%)*	*45/50 (90%)*	*5/50 (10%)*	*0*	≤ 0.01
*TC*	*39/50 (78%)*	*8/50 (16%)*	*3/50 (6%)*	*47/50 (94%)*	*3/50 (6%)*	*0*	*0.05*
*aTTL*	*29/50 (58%)*	*17/50 (34%)*	*4/50 (8%)*	*44/50 (88%)*	*6/50 (12%)*	*0*	≤ 0.01
*pTTL*	*35/50 (70%)*	*14/50 (28%)*	*1/50 (2%)*	*45/50 (90%)*	*5/50 (10%)*	*0*	*0.04*
*Spring ligament complex*							
smCNL	*38/50 (76%)*	*12/50 (24%)*	*0*	*48/50 (96%)*	*2/50 (4%)*	*0*	*0.01*
mpoCNL	*40/50 (80%)*	*10/50 (20%)*	*0*	*46/50 (72%)*	*4/50 (8%)*	*0*	*0.2*
iplCNL	*40/50 (80%)*	*10/50 (20%)*	*0*	*48/50 (96%)*	*2/50 (4%)*	*0*	*0.03*
*Talonavicular ligament*	*36/50 (72%)*	*13/50 (26%)*	*1/50 (2%)*	*50/50 (100%)*	*0*	*0*	≤ 0.01

Additional figures are given in the Supplementary Material (Figs. S1–S4).

##### Extent of talar head edema 

The volume of the bone marrow edema in the talar head was on average 1.7 ml (SD ± 4). There was no significant correlation between the number of ligamentous injuries and edema size (*p* = 0.5), with a correlation coefficient of *r* = 0.09. Similarly, there was no significant correlation regarding the size of bone marrow edema and number of injured compartments (*p* = 0.5), with a correlation coefficient of *r* = 0.1.

#### Cartilage injuries

There were no talar dome cartilage injuries.

#### Further workup and treatment

In our study, seven patients (14%) with medioplantar bone edema showed impingement syndrome in the course of time (1–12 months after trauma). Ligament reconstruction was necessary in eight patients (16%) (1–16 months after trauma).

##### Interobserver agreement

Interobserver agreement for evaluation of ligamentous injury was moderate to excellent (*κ* = 0.521–0.955). There was excellent agreement regarding the presence or absence of bone marrow edema (*κ* = 1) and regarding the size of the bone marrow edema (ICC: 0.98, 95% confidence interval: 0.97–0.99).

## Discussion

The aim of our study was to investigate the predictive value of THE in acute ankle sprain for the presence of concomitant ligamentous injury of the ankle. Our data showed that the presence of THE is associated with more extensive ligamentous injury, in particular of the medial and lateral collateral ligament complex.

Bone marrow edema secondary to bone impaction or bone contusion is thought to represent trabecular microcontusions and fractures, edema, hemorrhage, or reaction after stress [6; 7]. Isolated edema of the talar head in patients with ankle sprains was not yet described in the literature so far so the intent of our study was to focus on isolated bone marrow edema of the talar head.

Gorbachova et al. reported an association of plantar talar head injuries with other osseous foot injuries, e.g., anterior calcaneus process or cuboid fractures. They hypothesized that the injury resulted from talo-cuboid and/or talo-calcaneal impaction from a supination injury of the foot and ankle [9]. In their study with 41 patients with talar head injury, no statistically significant association between talar head injuries and ruptures of the medial and lateral collateral ligament complex was found, but there was a significant correlation between talar head injuries and spring ligament complex injuries, probably because their study group comprised mostly patients with more extensive foot trauma and different trauma mechanisms [9]. In our study with patients with isolated bone marrow edema of the talus, we did find a statistically significant association between THE and injury of the medial and lateral collateral ligament complex. We also found a significant association between THE and talonavicular ligament (TNL) injuries, whereas the TNL was not investigated by Gorbachova et al.

Apart from acute trauma, there are many other reasons for bone marrow edema, such as stress reactions and altered biomechanics [10]. Bone marrow edema can be a very sensitive sign in impingement, as a result of posttraumatic changes, with anterior ankle impingement as one of the most common impingement types. But in those cases, bone marrow edema is usually seen in a specific location typical for the pathomechanism, like on the talar neck and anterior distal tibia [11; 12].

Also, bone marrow edema is commonly present after prolonged running [11] which is probably more a result of accumulation of micro-trauma or mechanical overload due to altered biomechanics [12]. In this case, we often see stress reactions or fractures of the tarsal bones [12]. There is no information in the literature about isolated finding in the talar head so far [13]. Further reasons, not trauma-associated, include immobilization, complex regional pain syndrome, bone infarcts, osteoarthritis, inflammatory arthritis, neuroarthropathy, and transient osteoporosis, often seen with more subchondral diffuse and multifocal distribution [7; 16; 17], not as in our study with typical localized edema.

The clinical prognosis of bone bruises seems generally good, with a normalization of MRI appearance within 2–3 months after trauma [14]. Treatment is non-operative and functional in the majority of cases with injuries of the lateral ankle ligaments with good to excellent functional outcome after 1 year [19; 20], whereas surgical management of other concomitant ligament lesions may be necessary [14]. After an ATFL injury for example, most patients can regain satisfactory function through conservative therapy and rehabilitation [18; 21].

Finally, most ankle ligament injuries are uncomplicated. As described in the “Results” section, most patients in our study did not need subsequent ligament reconstruction, and most patients did not develop ankle impingement syndrome.

Numerous articles have been published about ankle sprains with injury of the lateral and medial collateral ligaments [15–18]. TNL injuries have been underreported in the literature, but they are surprisingly common, with a recent prospective ultrasound study reporting 21% of patients with TNL injury after ankle sprains [19]. The TNL is an important stabilizer at the dorsal aspect of the talonavicular joint and bears tensile stress during walking. Its complete tear causes increased displacement of the talonavicular joint when under stress and may be implicated in later joint arthritis and foot and ankle symptomatology [19]. Our study confirms that TNL injuries are more common in patients with ankle injuries than initially thought: 14 out of 50 patients (28%) in the THE group had a TNL injury, whereas no patients in the control group had a TNL injury, suggesting a strong correlation with the pathomechanism of the injury.

Looking at the number of injured ligaments in total and also at the number of involved main compartments, we could show that patients with THE had significantly more injuries than patients without bone marrow edema. However, there was no correlation between the size of edema and the number of injured ligaments or compartments. Other or multiple foci of edema were described in recent studies [10; 15], so we focused on bone marrow edema of the talar head as an isolated finding. 

Regarding the mechanism of injury in patients with focal talar head bone marrow edema, we hypothesize that rupture of the anterior talofibular ligament and the anterior tibiotalar ligament as the most commonly injured ligaments may result in instability of the joint which induces more stress to the talar head. A possible theory why injuries of the spring ligament complex were less common in patients with edema of the talar head than for example injuries of the lateral complex is that the spring ligament is a very strong ligament, which stays intact more commonly than the lateral ligaments.

Our study has limitations. The exact trauma mechanism was given just in a few subjects; hence, the potential influence of a specific trauma mechanism (e.g., inversion vs. eversion) could not be investigated. Unfortunately, the exact date of accident was not well documented for all subjects. While in most patients the injury may have occurred within a couple of months prior to the MR examination, for some patients, the time between injury and examination may have been longer. Also, no information was available regarding prior or repetitive injuries before the current trauma. Finally, traumatic insult to bone can take the form of a single excessive high-impact force which is probably more an acute, stressful event or repetitive below-trauma threshold loads, either of which can cause overload of the tissue resulting in damage [20]. The difference in time from trauma suggests that the origin of edema remains unclear. The alterations in bone could be considered a direct posttraumatic consequence, as suggested, but also a mechanical overload or imbalance as another possible cause cannot be ruled out, especially in patients in which the trauma happened longer ago.

In conclusion, a bone marrow edema of the medioplantar talar head is indicative of extensive ligament injuries and a sign of severe ankle trauma. While the presence of bone marrow edema may be non-specific [21], it is a sensitive sign to suspect additional pathology and may act as a guide to correct interpretation of the MRI examination. This specific location of bone marrow edema can serve as useful differential diagnostic clue in ankle MRI. It draws attention to possible mechanisms or specific soft tissue injuries, such as injuries to the talonavicular ligament.

## Supplementary Information

Below is the link to the electronic supplementary material.

Supplementary file1 (DOCX 10085 kb)

## Abbreviations

ATFL: Anterior talofibular ligament
aTTL: Anterior tibiotalar ligament
CFL: Calcaneofibular ligament
FS: Fat saturated
ICC: Intraclass correlation coefficient
iplCNL: Inferoplantar longitudinal calcaneonavicular ligament
LC: Lateral compartment
MC: Medial compartment
mpoCNL: Medioplantar oblique calcaneonavicular ligament
PACS: Picture archiving and communication system
PTFL: Posterior talofibular ligament
pTTL: Posterior tibiotalar ligament
RIS: Radiology information system
SLC: Spring ligament complex
smCNL: Superomedial calcaneonavicular ligament
STIR: Short tau inversion recovery
TE: Echo time
TCL: Tibiocalcaneal ligament
THE: Talar head edema
TNL: Talonavicular ligament
TR: Repetition time
TSL: Tibiospring ligament
