# Supplementary material for: Bone marrow edema of the medioplantar talar head is associated with severe ligamentous injury in ankle sprain
Source: Skeletal Radiol. 2022 Mar 31;51(10):1937–46. doi: 10.1007/s00256-022-04043-3 (PMC9381494; doi:10.1007/s00256-022-04043-3)
Supplement: Supplementary file 1 — Supplementary file1 (DOCX 10085 kb) [file 256_2022_4043_MOESM1_ESM.docx]

**Supplementary Material**

**Additional Images**


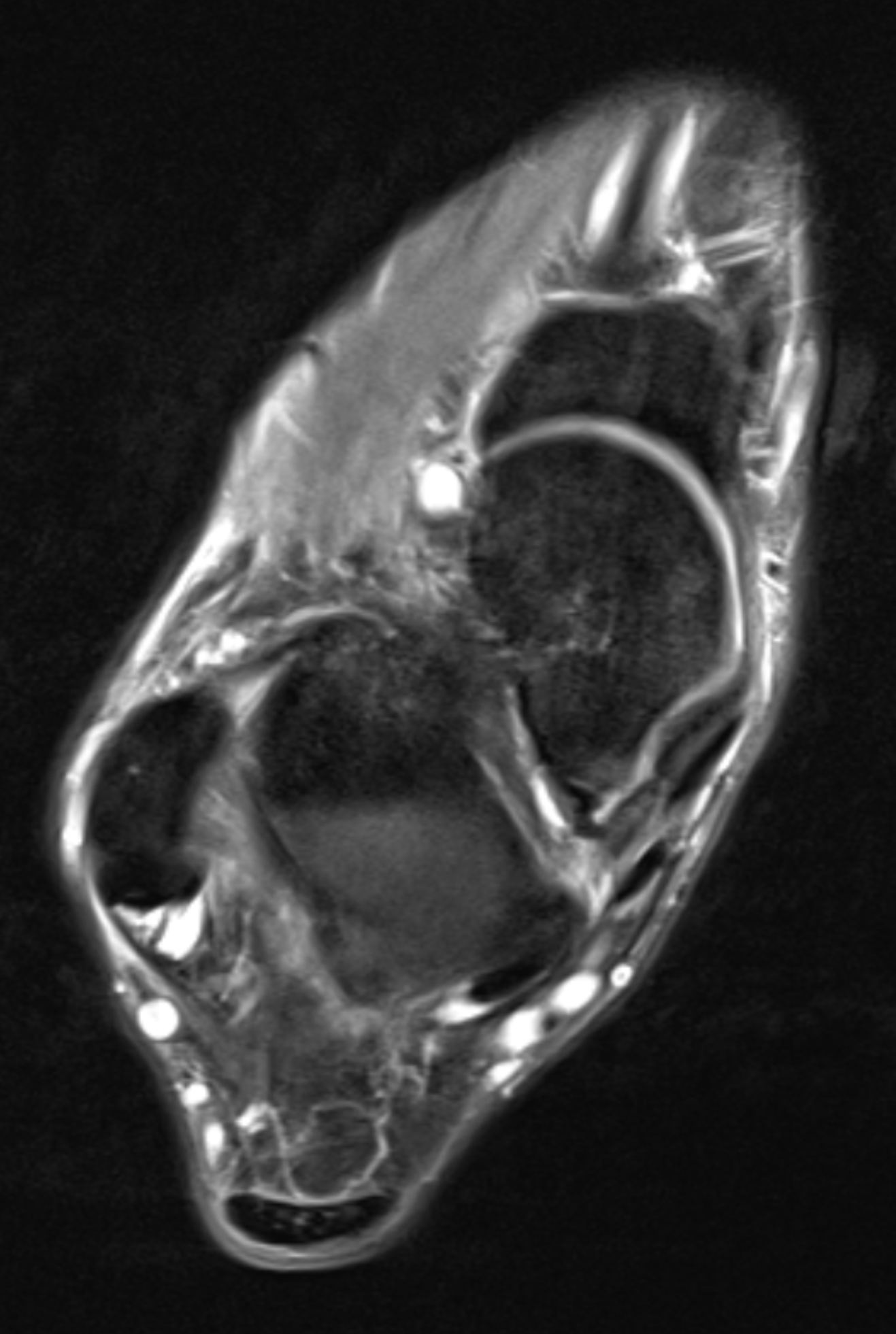

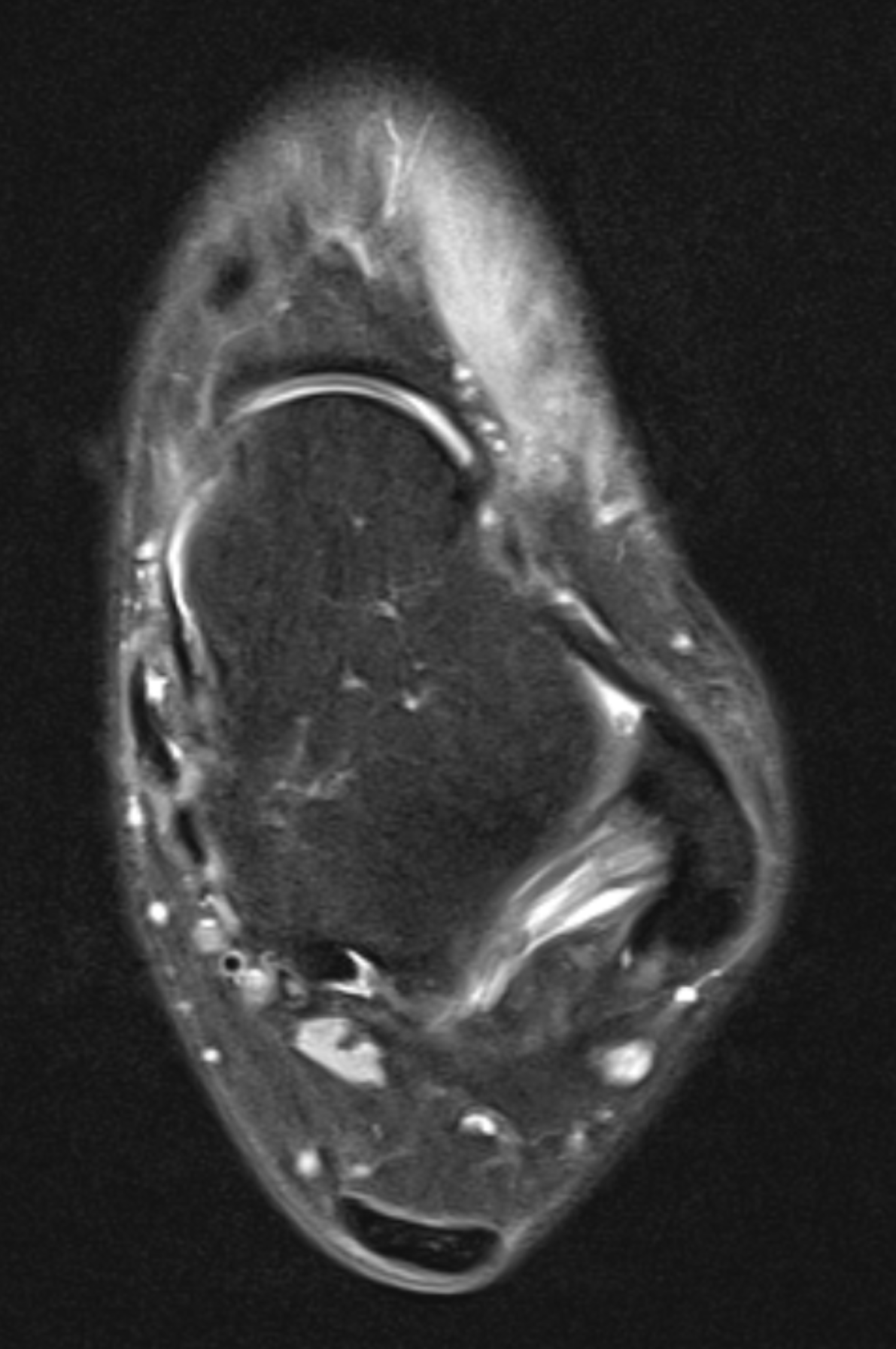


a b

**Fig. S1** 19-year-old man with ankle distorsion 9 days before imaging (a). Axial Proton Density fat saturated images showed sprain of the anterior talofibular ligament (black arrow) with hyperintense signal and partial discontinuity.

In comparison, a normal anterior talofibular ligament (white arrow) of a patient about the same age (b).


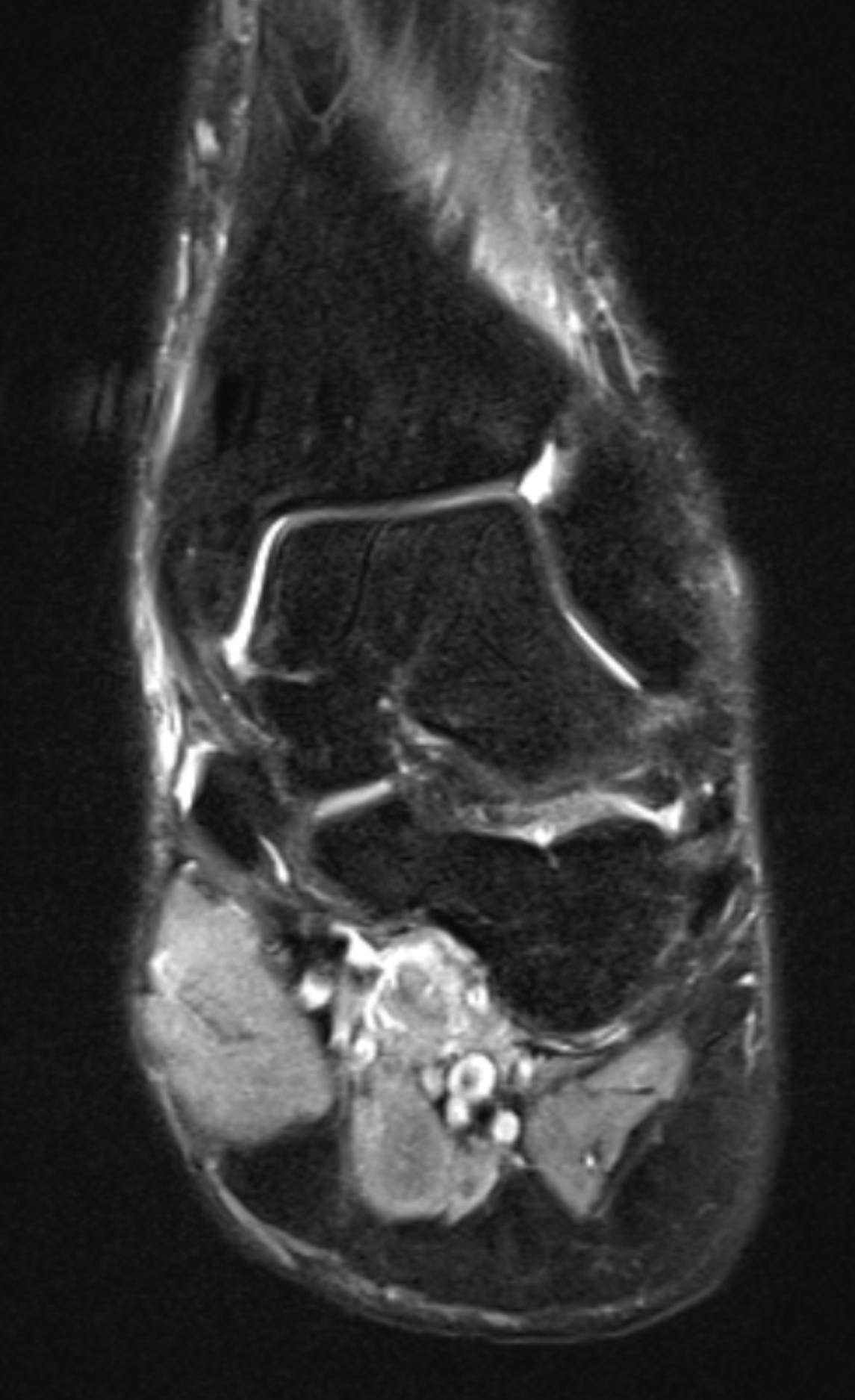


**Fig. S2** 35-year-old woman with ankle distorsion two weeks before. Coronal Proton Density fat saturated images showed an intact anterior tibiotalar ligament with no fiber disruption and dark signal (arrow).

b


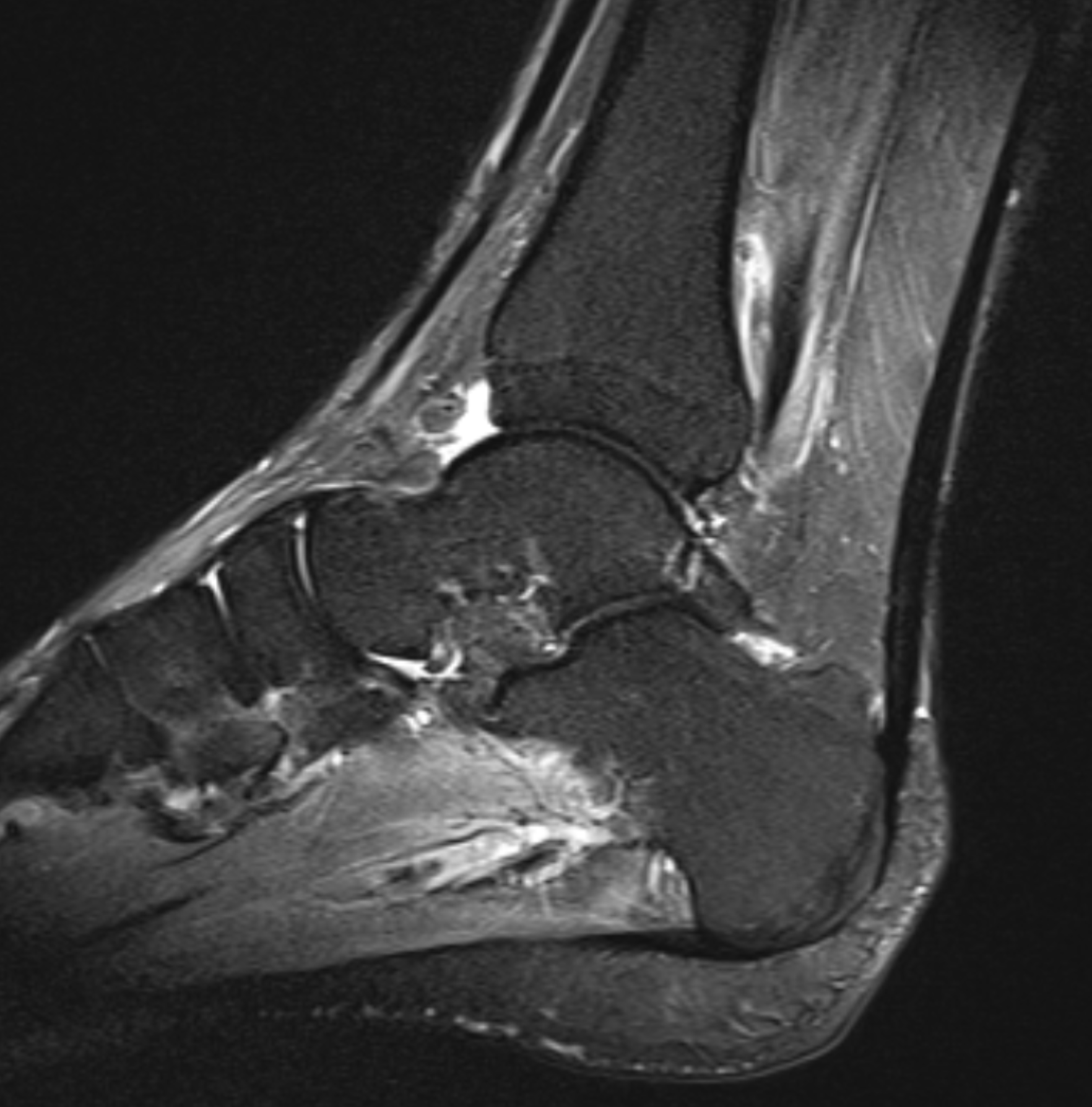


**Fig. S3** 42-year old woman with supination trauma 10 days before. Sagittal Short Tau Inversion Recovery sequence showed a normal talonavicular ligament (arrow).

a


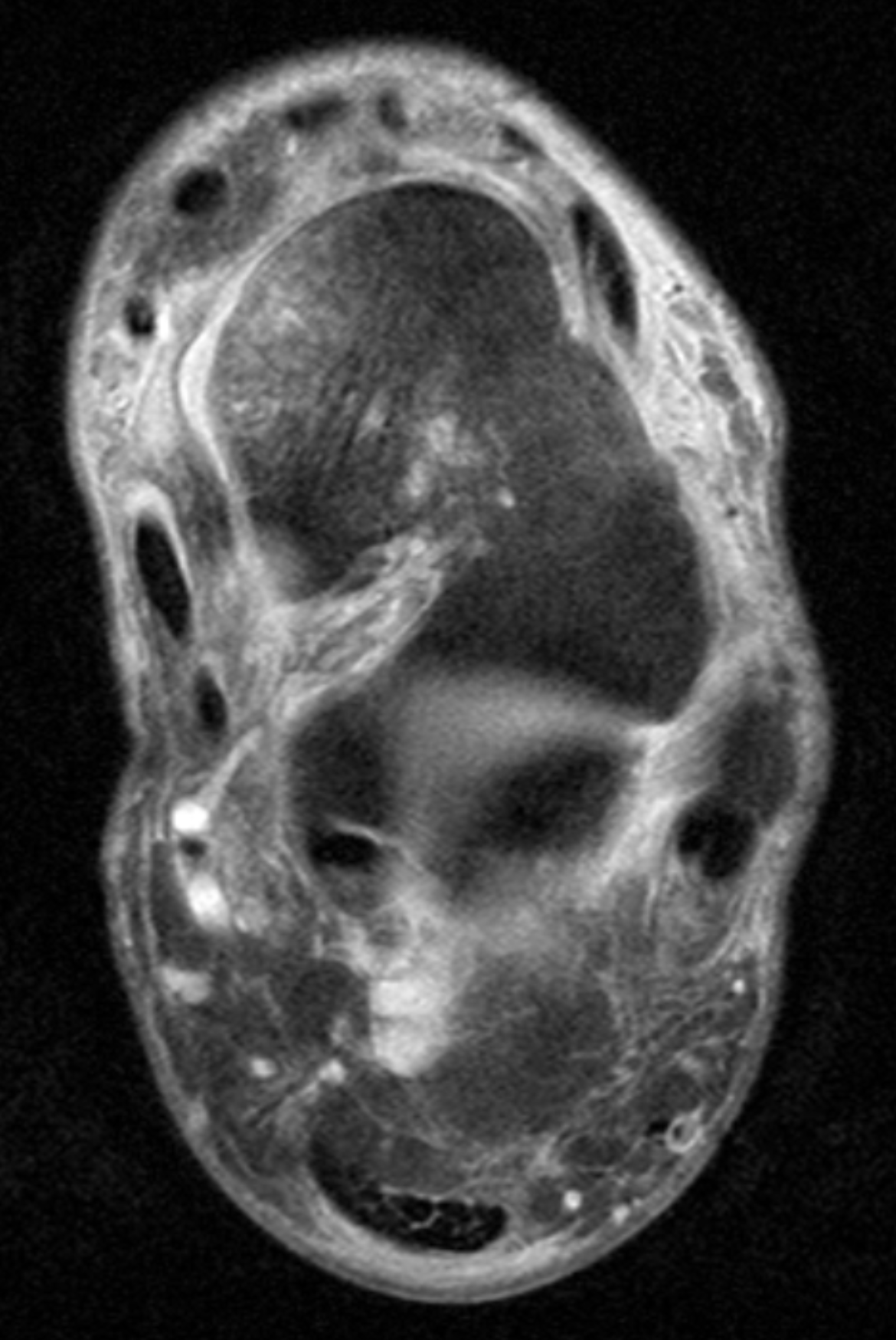


**Fig. S4** 54-year-old woman with ankle trauma six weeks before imaging. Axial Proton Density fat saturated images showed complete rupture of the springligament (white arrow), associated with moderate edema of the medioplantar head of the talus (black arrow).
